# Morphology, Activation, and Metal Substitution Effects of AlPO_4_-5 for CO_2_ Pressure Swing Adsorption

**DOI:** 10.3389/fchem.2020.568669

**Published:** 2020-10-06

**Authors:** Andreas Papageorgiou, K. Suresh Kumar Reddy, Dimitrios Karonis, Donald Reinalda, Yasser Al Wahedi, Georgios N. Karanikolos

**Affiliations:** ^1^Department of Chemical Engineering, Khalifa University, Abu Dhabi, United Arab Emirates; ^2^School of Chemical Engineering, National Technical University of Athens, Athens, Greece; ^3^Center for Catalysis and Separations (CeCaS), Khalifa University, Abu Dhabi, United Arab Emirates; ^4^Research and Innovation Center on CO_2_ and H_2_ (RICH), Khalifa University, Abu Dhabi, United Arab Emirates

**Keywords:** aluminophosphates, adsorption, carbon dioxide, AFI, metal substitution, zeolites, adsorption kinetics, PSA

## Abstract

Aluminophosphate, AlPO_4_-5, an AFI zeotype framework consisting of one-dimensional parallel micropores, and metal-substituted AlPO_4_-5 were prepared and studied for CO_2_ adsorption. Preparation of AlPO_4_-5 by using different activation methods (calcination and pyrolysis), incorporation of different metals/ions (Fe, Mg, Co, and Si) into the framework using various concentrations, and manipulation of the reaction mixture dilution rate and resulting crystal morphology were examined in relation to the CO_2_ adsorption performance. Among the various metal-substituted analogs, FeAPO-5 was found to exhibit the highest CO_2_ capacity at all pressures tested (up to 4 bar). Among the Fe-substituted samples, xFeAPO-5, with x being the Fe/Al_2_O_3_ molar ratio in the synthesis mixture (range of 2.5:100–10:100), 5FeAPO-5 exhibited the highest capacity (1.8 mmol/g at 4 bar, 25°C) with an isosteric heat of adsorption of 23 kJ/mol for 0.08–0.36 mmol/g of CO_2_ loading. This sample also contained the minimum portion of extra-framework or clustered iron and the highest mesoporosity. Low water content in the synthesis gel led to the formation of spherical agglomerates of small 2D-like crystallites that exhibited higher adsorption capacity compared to columnar-like crystals produced by employing more dilute mixtures. CO_2_ adsorption kinetics was found to follow a pseudo–first-order model. The robust nature of AlPO_4_-5–based adsorbents, their unique one-dimensional pore configuration, fast kinetics, and low heat of adsorption make them promising for pressure swing adsorption of CO_2_ at industrial scale.

## Introduction

The severe environmental concern related to the greenhouse effects is mainly attributed to gases that absorb and emit radiation within the thermal infrared range. The primary greenhouse gases are carbon dioxide, methane, water vapor, nitrous oxide, and ozone. CO_2_ is the most important contributor coming mainly from combustion of fossil fuels and being released into the atmosphere from the power plants, steel plants, cement industry, and other large-scale industrial operations, as well as from transportation (Kontos et al., 2014). Notably, the increase of total primary energy supply, reaching 150% in 2014 compared to 1971, is caused by the associated worldwide economic growth with fossil fuels holding the most significant share for that energy produced (Metz et al., 2005; IEA, 2016; Tabish et al., 2020). Under these circumstances, it is evident that carbon dioxide emissions will keep increasing as energy demands will continue being covered primarily by fossil fuels at least for the next 50 years.

CO_2_ capture, utilization, and storage (CCUS) technologies have been proposed to stabilize the concentrations of greenhouses gases in the atmosphere and mitigate their impact. Among other proposed solutions, such as retrofits of existing units, usage of fuels with less carbon dioxide footprint, and nuclear and renewable energy, CCUS is the most promising one in terms of compatibility with the energy production, continued dependence on fossil fuels, and delivery infrastructure. Focusing on postcombustion, different technologies have been used. Employing amine-based solvents is the most mature technology. Monoethanolamine, diethanolamine, and *N*-methyldiethanolamine are commonly used alkanolamines. These lean amines, commonly diluted along with water of content in the order of about 70%, have a high reactivity toward CO_2_. Still, the technology has certain drawbacks mainly associated with high energy consumption for the regeneration step, requirement of large voluminous equipment, high corrosion rates, and solvents slippage to the atmosphere (Resnik et al., 2004; Haszeldine, 2009). As promising alternatives to solvent-based systems, which have constituted the main industrial practice for several decades, technologies based on adsorption and membrane separation are gaining considerable attention (Pilatos et al., 2010; Labropoulos et al., 2015; Kueh et al., 2018). Concerning adsorption, emphasis is put on porous materials. These can be divided into two categories: (i) physical adsorbents, such as porous carbons, zeolites, and metal–organic frameworks, and (ii) chemical adsorbents, such as functionalized materials with surface agents such as amine moieties (Choi et al., 2009; Sayari et al., 2011; Pokhrel et al., 2018; Varghese and Karanikolos, 2020). In this front, various materials are being discovered and explored, yet a platform of suitable materials/systems to treat a wide range of industrial emissions at a large scale still remains a challenge. The reason is that multiple factors need to be met at the same time, i.e., adsorbents need to exhibit (i) high capacity; selectivity compared to other components such as NO_x_, SO_2_, and H_2_O vapor; fast adsorption/desorption kinetics; and low energy consumption; and (ii) chemical and thermal stability, sustainable performance for many cycles, low manufacturing cost, and mechanical robustness at large scale.

Zeolites have been studied for CO_2_ capture, particularly involving dry CO_2_, based on their relatively high adsorption capacity (Chue et al., 1995; Sircar and Golden, 1995; Siriwardane et al., 2001; Chou and Chen, 2004), low-cost of production, and excellent thermal stability (Musyoka et al., 2015). Yet, due to polar zeolite surfaces, these materials are highly hydrophilic resulting in lower CO_2_ capture capacity and selectivity and early saturation in the presence of moisture (Corma, 2003). To battle this problem, less hydrophilic zeolite-type materials need to be explored. Aluminophosphates (AlPOs) are among the most notable adsorbents for this duty. AlPOs were discovered in 1982 by Wilson and partners of Union Carbide (Wilson et al., 1982). The framework of AlPOs, such as AlPO_4_-5, consists of alternating Al^3+^ and P^5+^ connected by oxygen atoms (Wilson et al., 1982; Stoeger et al., 2012). The main feature is the charge neutrality of the framework, which occurs from the equal ratio of alumina to phosphorus (Al/P = 1). This constant ratio producing materials of a net neutral electric charge prevents ion exchange, and as a result, AlPOs tend to be only slightly hydrophilic due to the absence of acidic sites (Cundy and Cox, 2003; Carmine, 2012). Indeed, in our recent work, we showed that AlPO_4_-5 is rather hydrophobic particularly at relatively low water partial pressures, where water molecules occupy niches close to pore walls, followed later by the filling of the central pore area (Schlegel et al., 2018). However, AlPOs retain their CO_2_ sorption capacity potential due to the high concentration of physisorption sites. Therefore, the limited hydrophilicity, concentration of physisorption sites, and more linear shape of the CO_2_ adsorption isotherms as compared to other zeolites suggest that these materials would have a longer process lifetime extending over many adsorption/regeneration cycles by pressure-mediated tuning between adsorption/desorption cycles [pressure swing adsorption (PSA)]. Liu et al. (2011) examined 8-member ring AlPOs (AlPO_4_-17, AlPO_4_-18, AlPO_4_-53, and AlPO_4_-25) at different temperatures and determined CO_2_ uptake capacities in the range of 1.52–2.32 mmol/g at 273–293 K and 100 kPa. Among the above structures, AlPO_4_-53 possessed a higher CO_2_ affinity and a CO_2_/N_2_ selectivity of 98.4 with low interaction with water molecules as compared to the benchmark zeolite 13X. Zhao et al. (2009) investigated AlPO_4_-14 and reported CO_2_ adsorption capacities ranging from 2.0 to 2.7 mmol/g within the temperature range of 273–300 K at 100 kPa, exhibiting also relatively high CO_2_ over CH_4_ selectivity. Delgado et al. (2013) studied the adsorption behavior of AlPO_4_-11 for CO_2_, CH_4_ and N_2_ and reported a CO_2_ adsorption capacity of 0.31-0.7 mmol/g at 298–338 K and 100 kPa.

In addition to high adsorption capacity and selectivity, fast adsorption/desorption kinetics and a low heat of adsorption are key factors for an industrially prominent adsorbent candidate in PSA CO_2_ capture applications. The work reported herein studies the synthesis and modification/functionalization of AlPO_4_-5 and its metal substituted analogs as potential candidates for CO_2_ capture. The crystal lattice of AlPO_4_-5 possesses a hexagonal symmetry and a monodirectional channel morphology extending along the *c*-axis. The main channels are created by 12-member rings of alternating tetrahedra of [AlO_4_]^−^ and [PO_4_]^+^ having a diameter of 7.2 Å (Rajic and Kaucic, 2002; Guo et al., 2005; Karanikolos et al., 2008). Here, we assess the impact of various factors on CO_2_ sorption, namely, (a) the effect of various heteroatoms used in isomorphic substitution in the AlPO_4_-5 framework at varying concentrations, (b) the pore activation method and in particular the affinity of remnant carbon species present into the inner pore surface after structure-directing agent (SDA) removal by two different thermal treatment methods, i.e., partial oxidation and pyrolysis, and (c) the impact of AlPO_4_-5 crystal morphology and hydrothermal synthesis mixture composition and in particular the water content in the mixture.

## Experimental Section

### Materials

Aluminum isopropoxide (Merck), orthophosphoric acid (85% in H_2_O, Sigma Aldrich), and triethylamine (TEA, Merck) were used as precursors for AlPO_4_-5 growth. Tetraethyl orthosilicate (Merck), magnesium chloride (Merck), cobalt (II) acetate tetrahydrate (98%, Sigma-Aldrich), and iron (III) nitrate non-ahydrate (Merck) were used as metal precursors for the synthesis of the metal-substituted AlPOs (MeAPO-5).

### Growth of AlPO_4_-5 and MeAPO-5 Adsorbents

The AlPO materials were grown hydrothermally from reaction mixtures starting from a gel composition of 1Al_2_O_3_:1.3P_2_O_5_:1.2TEA:*x*Me:*y*H_2_O, where *x* and *y* refer to the metal/Al_2_O_3_ and water/Al_2_O_3_ molar ratios, respectively. Desired amount of aluminum isopropoxide was dissolved in deionized water under stirring for 3 h. To this solution, orthophosphoric acid was added dropwise, and the mixture was stirred for another 1 h. TEA, as the SDA, was then added dropwise, and the mixture was stirred for 24 h. For the preparation of the metal or ion substituted AlPOs, the metal precursor was added right after the addition of the SDA. The reaction gel having a pH ranging from 5 to 6 was transferred to a Teflon-lined stainless steel autoclave and placed inside a preheated oven at a temperature of 160°C for 24 h. After growth, the autoclave was quenched, and the solid product was collected after repeated centrifugation/washing cycles. The obtained crystals were dried at 80°C for 6 h and were subsequently calcined in air using a ramping rate of 2.5°C/min until temperature reached 80°C, keeping temperature stable for 30 min there, and further increasing it up to 600°C, where it was kept constant for 5.5 h. Activation at various temperatures under air flow (calcination) or nitrogen (pyrolysis) in a tubular furnace was also performed in order to parametrically explore decomposition/removal of the SDA occluded into the pores using the above temperature ramping program. A water/Al_2_O_3_ molar ratio of 100:1 was used in these experiments. The MeAPOs were synthesized using different metals/ions (Fe, Mg, Co, and Si) into the AlPO_4_-5 framework with water/Al_2_O_3_ molar ratio of 100:1 and metal/Al_2_O_3_ ratio of 5:100. For the FeAPO-5 adsorbents, additional metal contents were studied as well.

The following naming code of samples was applied throughout the various sets of experiments in this work:

For the activation set of experiments: *T*AlPO_4_-5.P and *T*AlPO_4_-5.C, where *T* is the thermal treatment temperature in °C, and P and C stand for pyrolysis under inert atmosphere and calcination under airflow, respectively.

For the metal substitution set of experiments: The molar ratios were placed ahead of the sample names. For example, 5FeAPO-5 indicates a metal/Al_2_O_3_ ratio of 5:100.

For the experiments on varying water content in the synthesis mixture: The H_2_O/Al_2_O_3_ molar ratio was placed ahead of the sample names. For example, 400AlPO_4_-5 indicates a H_2_O/Al_2_O_3_ ratio of 400:1.

### Characterization

The crystallinity of the synthesized samples was investigated by X-ray diffraction (XRD) using a Panalytical X'Pert Pro Powder Diffractometer. A Cu-Kα monochromatized radiation source with wavelength λ = 1.5406 Å, power 40 kV, and current of 40 mA was utilized, and the scan speed was set to 0.02 degrees/s. Morphology evaluation was performed by scanning electron microscopy (SEM) using an FEI Quanta 200 microscope. Samples were placed on a carbon tape and were coated by gold to enhance the conductivity and to allow observation at 30 kV. Ultraviolet-visible diffuse reflectance spectroscopy (UV-Vis DRS) of the adsorbents was recorded using a Varian Analytical Cary 5000 UV-Vis-NIR spectrometer, equipped with diffuse reflectance accessory used to record electronic spectra from 200 to 800 nm. Thermogravimetric analysis (TGA) was performed using TA instruments Trios V3.1 analyzer under 50 mL/min of air flow with a ramping rate of 10°C/min from 25 to 900°C. Fourier-transform infrared spectroscopy (FTIR) was carried out on a BRUKER TENSOR II series FTIR spectrometer with the aid of diamond attenuated total reflectance crystal, where thin sections of the samples were scanned in the wavenumber region of 4,000 to 400 cm^−1^ with a resolution of 4 cm^−1^ by undergoing 32 scans. Nitrogen adsorption–desorption analysis was performed at 77 K using a Micromeritics 3-Flex analyzer. Before the analysis, the samples were degassed at 220°C for 3 h. The surface area was determined by the Brunauer–Emmett–Teller (BET) method. Pore size distribution and pore volume were obtained by processing adsorption–desorption data using the Barrett, Joyner, Halenda (BJH) and Horvath–Kawazoe (HK) models via the MicroActive software.

### CO_2_ Adsorption

CO_2_ adsorption experiments were carried out using a Rubotherm gravimetric sorption analyzer (IsoSORP STATIC 3xV-MP). The magnetic suspension balance measures sample weight and gas dosing to determine the adsorption equilibrium. Approximately 50 mg of each activated adsorbent was first heated under vacuum at 150°C for 3 h to remove moisture and other volatile substances until a constant sample mass was obtained. Buoyancy correction was performed using helium gas and incorporated into the sample weight. The sample in a stainless-steel holder was pressurized with CO_2_ gas up to 4 bar (in steps of 1 bar), and continuous measurement of sample mass gain during CO_2_ adsorption was determined at 25°C under equilibrium/saturation conditions. The gravimetric analyzer software uses the recorded mass changes to estimate the sorption capacity at each pressure step and incorporates the buoyancy correction as well in order to generate the final CO_2_ sorption isotherm. For the adsorption kinetics study, the adsorbents were measured again but with a single step directly up to 4 bar taking capacity values at regular time intervals. The procedure was repeated for three different temperatures, namely, 25, 45, and 60°C.

## Results and Discussion

### Activation of Adsorbents by Calcination and Pyrolysis

Upon growth of porous crystalline materials such as zeolites, SDAs (or organic templates) are typically used to direct the formation of the pores. Following growth, removal of these molecules from the pores needs to take place as to activate the open porosity of the materials. Whether the occluded molecules are completely decomposed and removed or remnants of carbon still exist into the pores may affect the affinity and interaction of the internal surface with CO_2_. This was first investigated in our study by activating the resulting AlPO_4_-5 crystals via calcination and partial calcination (air flow) or pyrolysis (nitrogen atmosphere) at various temperatures. The latter was employed based on our previous work revealing that graphitic carbon in the form of carbon nanotubes (CNTs) into the pores of oriented AlPO_4_-5 films affected CO_2_ sorption, as well as permeance behavior through the resulting CNT membranes (Labropoulos et al., 2015). In addition, the occluded amine SDA is partially decomposed and removed upon partial calcination, which might create extra spacing/porosity within the larger AlPO_4_-5 channels. The objective was to examine whether remaining carbon species would act favorably toward CO_2_ adsorption via interaction of CO_2_ with both AlPO_4_-5 and carbon surfaces, or complete removal of SDA would be preferred due to maximizing the pore volume and carbon-free AlPO_4_-5 surface.

Consequently, for calcination, five different temperatures were used, namely, 300, 400, 500, 600, and 700°C under airflow. The distinct color of the materials produced at the different calcination temperatures is shown in Supplementary Figure 1. The as-synthesized AlPO_4_-5 crystals had a white color. The material calcined between 300 and 500°C had a distinct brown coloration that turned softer as the temperature increased, which indicates the existence of remaining carbon species at the external crystal surface at these relatively low calcination temperatures with carbon content being decreased with increase in temperature. At temperatures above 600°C the materials turned white again, indicating complete carbon removal.

Pyrolysis of the synthesized AlPO_4_-5 samples was carried out under nitrogen flow at temperatures of 240, 400, and 700°C. The aim was to investigate possible affinity enhancement effects between CO_2_ and carbon species resulting from the TEA pyrolysis in the pores or formation of additional carbon porosity/surface within the AFI channels. The creation of porous carbon-based structure and possibly extra porosity inside the channels of AlPO_4_-5 might enhance affinity toward CO_2_ due to the existence of pyrolytic carbon. Analogous treatment yielded formation of single-wall CNTs inside the AFI pores of powder AlPO_4_-5 crystals (Tang et al., 1998), as well as in oriented membrane configuration for membrane-based gas separation, as demonstrated in our previous work (Labropoulos et al., 2015). The pyrolysis temperature of 240°C was selected as it represents a point within the TEA decomposition region based on TGA data (Wan et al., 2000).

**Figure 1:**
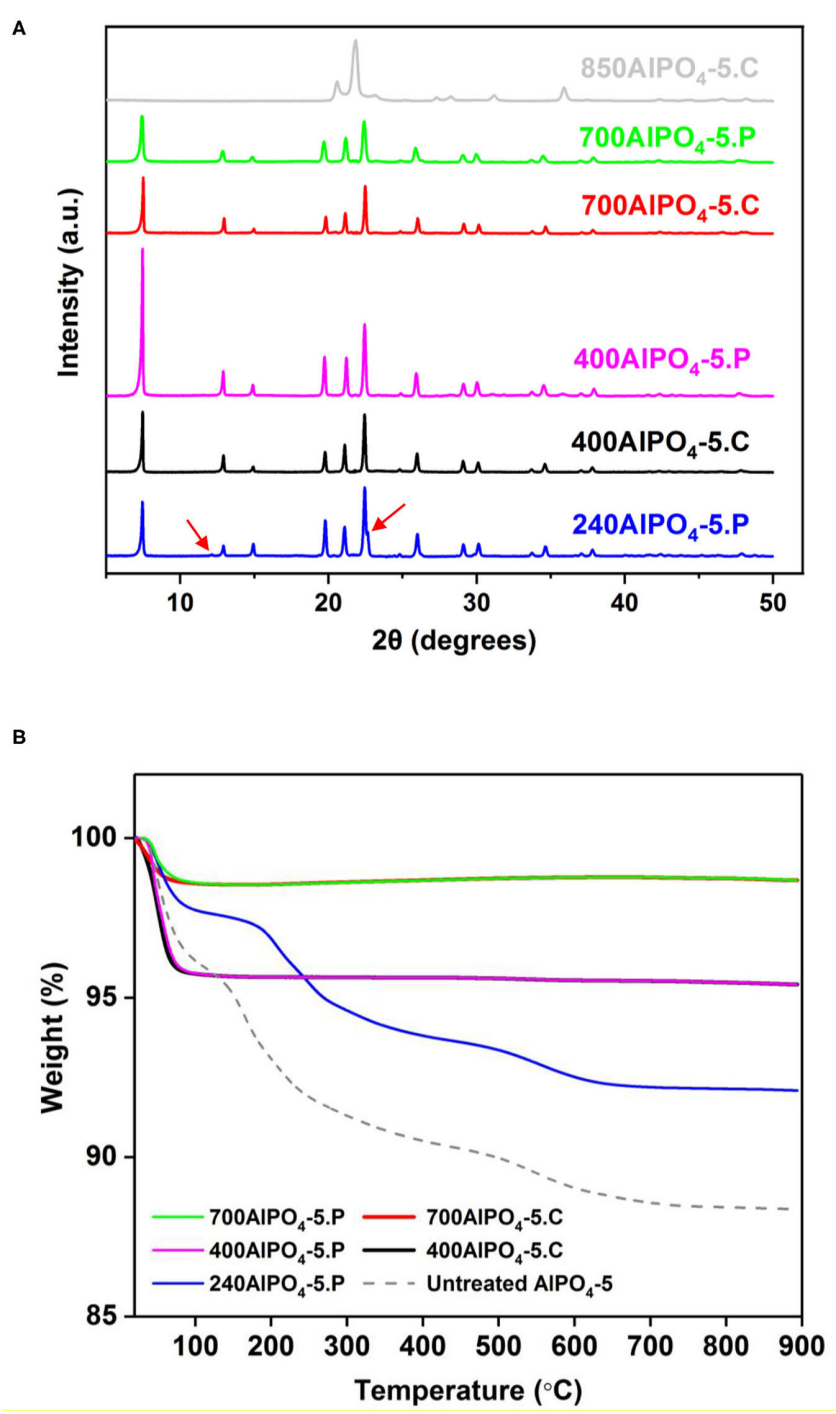
**(A)** XRD patterns and **(B)** TGA profiles of calcined and pyrolyzed AlPO_4_-5 adsorbents treated at different temperatures.

XRD analysis of selected calcined and pyrolyzed samples are shown in Figure 1A. The water/Al_2_O_3_ molar ratio was fixed at 100 for this activation effect study. All samples treated at temperatures up to 700°C exhibit the characteristic diffraction peaks of AFI (Karanikolos et al., 2008; Basina et al., 2018), confirming the stability and structural integrity of the materials up to this temperature. Notably, the sample pyrolyzed at the lowest temperature (240°C), in addition to the AFI peaks, exhibits an additional peak shoulder at 22.6° and a minor peak at 12.1°, which are attributed to remnant carbon clusters (Sawant et al., 2017). At tested temperatures of 400°C and above, the above peaks disappear indicating that the remnant carbon content is significantly reduced or eliminated. Furthermore, such peaks do not exist in the untreated AlPO_4_-5 material either (Supplementary Figure 2). Thermal stability of a potential adsorbent is one of the key quality features for industrial application ensuring robustness upon thermal stresses that may occur during preparation as well as application. e.g., upon regeneration or other processing steps. The high temperature treatment applied here and the obtained stability of the AlPO_4_-5 adsorbents confirm their robustness and suitability for high temperature application. In order to explore the upper temperature stability limit, we also calcined the material at 850°C. There, we observed a structure collapse and transformation into a dense AlPO_4_-tridymite phase (Stoeger et al., 2012).

The carbon content of the thermally treated samples and their corresponding thermal behavior in comparison to untreated AlPO_4_-5 was further studied by TGA, which was performed under oxidative conditions (air). According to the obtained results (Figure 1B), the pyrolyzed and calcined adsorbents exhibit almost the same TGA profiles as revealed for the corresponding samples treated at 400 and 700°C Notably, the only significant weight loss experienced by both of the above sets of samples is the one at the low temperature range of up to 100°C, which is attributed to removal of adsorbed moisture. The absence of any noticeable transition due to amine decomposition indicates that the occluded amine molecules have been almost completely removed from the pores at these temperatures. In addition, the samples treated at 700°C exhibit a more hydrophobic behavior containing 1.3 wt% of moisture compared to the ones treated at 400°C that contain approximately 4.2 wt% of moisture (Table 1). This is attributed to the fact that polar functional groups on the AlPO_4_-5 surface, such as Al-OH and P-OH (Peri, 1971), are being compromised by the higher temperature treatment, thus suppressing hydrophilicity. The untreated AlPO_4_-5 adsorbent and the one pyrolyzed at 240°C exhibit two distinct weight loss transitions after the removal of the adsorbed water. The first transition is attributed to decomposition of the TEA molecules from the pores and occurs in the temperature range of 120–300°C for the untreated sample, while it starts at a higher temperature (165°C) for the pyrolyzed one. This temperature difference is due to the fact that portion of the amine has already been decomposed in the latter sample. The second transition occurs at a considerably higher temperature (500–650°C) and is attributed to the removal of trapped and possibly graphitized carbon species from the pores. As per Table 1, the total organic content in the untreated sample is 7.8 wt%, whereas that of the sample pyrolyzed at 240°C is 5.66 wt%. The existence of remnant carbon in the latter sample is in agreement to XRD evidence discussed above.

**Table 1:** Characteristic TGA transitions and corresponding weight loss percentages for the various calcined and pyrolyzed AlPO_4_-5 adsorbents.

**Adsorbent**	**% Weight loss up to 100°C (adsorbed water)**	**% Weight loss between 100 and 900°C (organic content)**
700AlPO_4_-5.C	1.32	0.10
700AlPO_4_-5.P	1.32	0.07
400AlPO_4_-5.C	4.25	0.34
400AlPO_4_-5.P	4.24	0.35
240AlPO_4_-5.P	2.25	5.66
Untreated AlPO_4_-5	3.84	7.80

**Figure 2:**
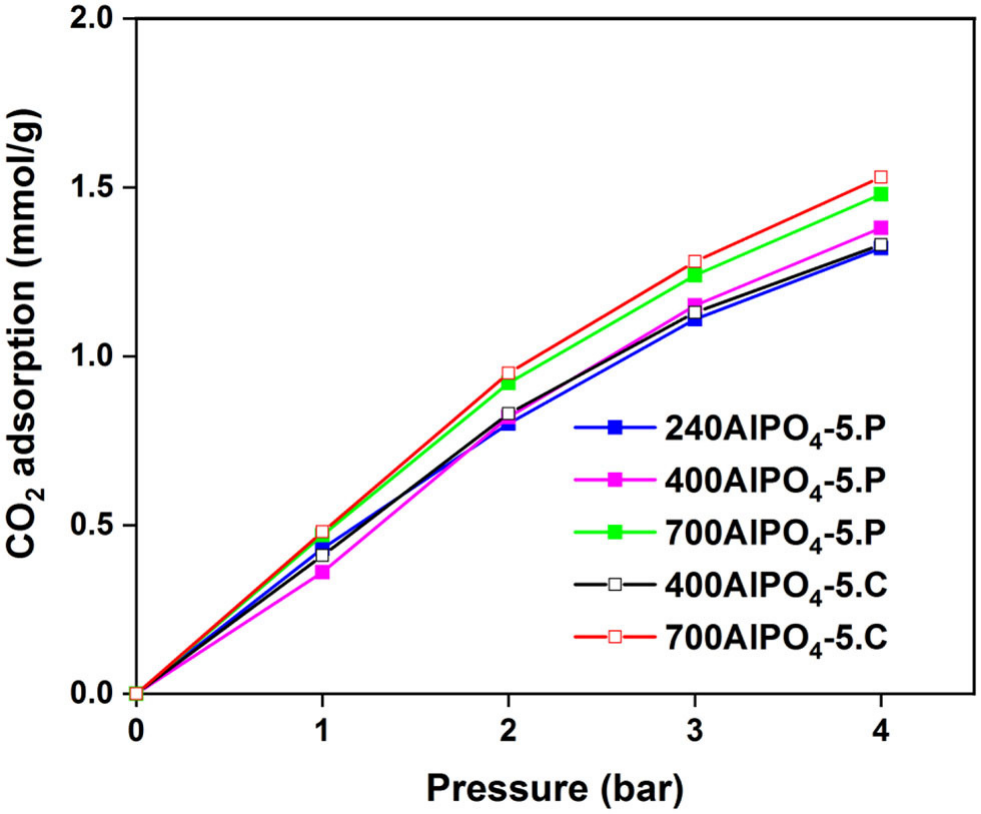
CO_2_ adsorption isotherms of calcined and pyrolyzed AlPO_4_-5 adsorbents at 25°C.

The effect of the calcination and pyrolysis treatment on the CO_2_ adsorption capacity is depicted in Figure 2 (data in Supplementary Table 1). The sample calcined at 700°C exhibits higher CO_2_ capacity (1.53 mmol/g at 4 bar) than the one calcined at 400°C. This observation is consistent throughout the pressure range examined (up to 4 bar) and indicates that maximizing porosity in the channels of the adsorbent is critical. The sample pyrolyzed at 700°C exhibits higher CO_2_ capacity compared to the other pyrolyzed samples throughout the pressure range of 0 to 4 bar, with a maximum capacity of 1.48 mmol/g at 4 bar. This is attributed to the total decomposition and removal of TEA from the framework, and the creation of clean pores in the zeolite to host the CO_2_ molecules. The remaining two isotherms corresponding to the lower pyrolysis temperature samples (240AlPO_4_-5.P and 400AlPO_4_-5.P) are of interest since an inversion in the CO_2_ adsorption capacity can be observed. Specifically, the sample which was treated at 240°C exhibits higher capacity at low pressures (up to 2 bar), whereas at higher pressures (2–4 bar) the sample treated at 400°C adsorbs more CO_2_. In addition, a noticeable observation with respect to low pressure CO_2_ capture application is that, up to 1 bar, the sample pyrolyzed at 240°C exhibits almost same capacity as the one pyrolyzed at the highest temperature tested, i.e., 700°C, which is also very close to the capacity observed for the calcined sample at 700°C. This behavior indicates that the carbon species remnants into the pores created by low temperature SDA pyrolysis interact efficiently with CO_2_ at low pressures, whereas capture capacity at higher pressures is more favored by the increased pore volume and carbon-free surface created upon higher temperature thermal treatment. Conclusively, if the AlPO_4_-5 adsorbents are to be used for low-pressure CO_2_ capture, high thermal activation is not required, as similar capture capacity can be achieved by pyrolysis treatment at significantly lower temperatures, e.g., 240°C, thus saving energy and safeguarding the thermal stability of the adsorbents. The relatively high capacity resulting from such pyrolysis-based activation treatment is attributed to enhanced interaction of CO_2_ at low pressures with carbon species that are remnant in the pores upon partial SDA decomposition.

### Effect of Metal Substitution

**Figure 3:**
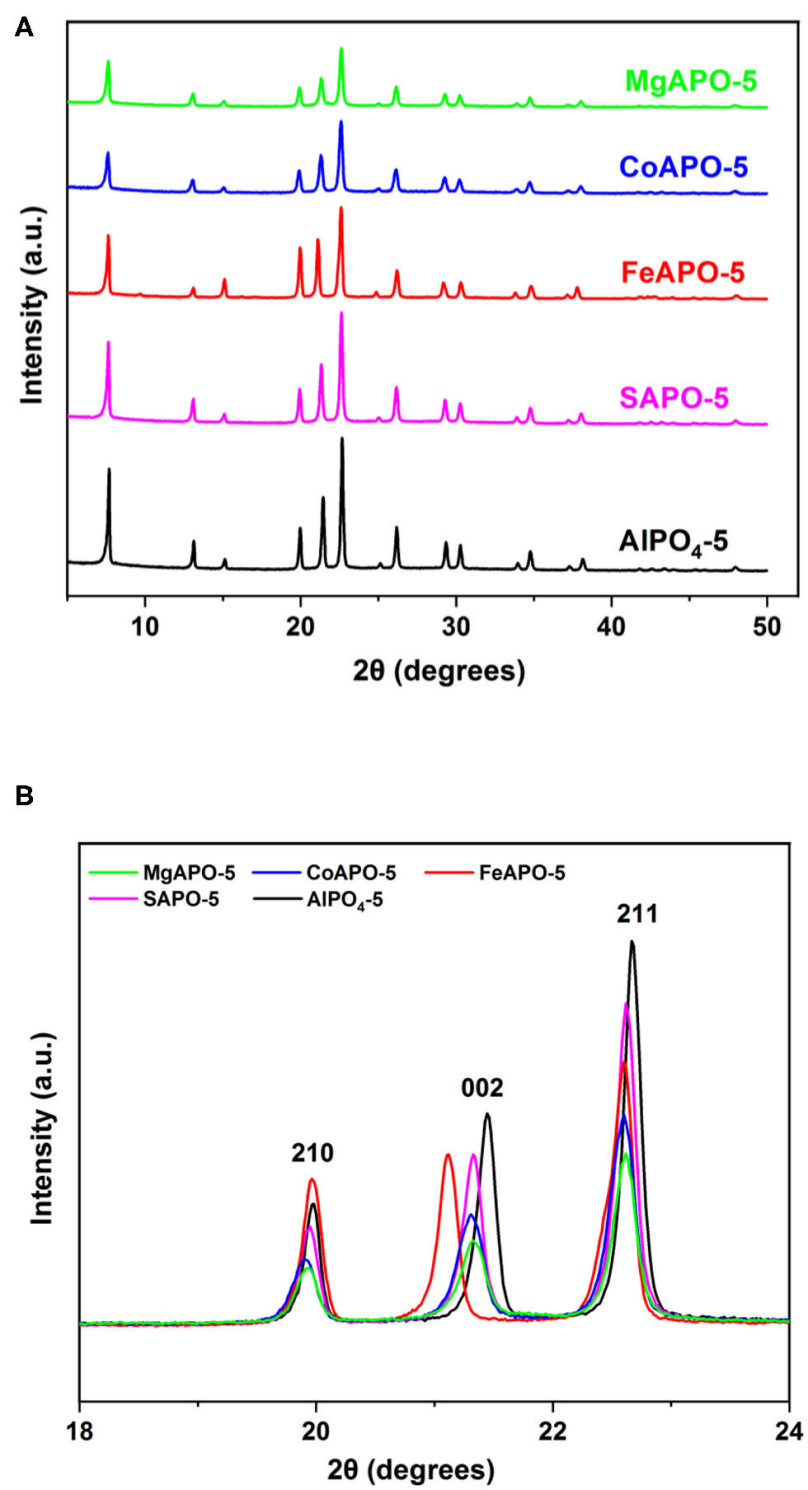
**(A)** XRD patterns of MeAPOs with Me/Al_2_O_3_ molar ratio in the synthesis mixture of 5:100, and **(B)** closer view of the AFI characteristic peaks between 19 and 23°.

Ion-substituted AlPO_4_-5 were prepared by incorporating Fe, Mg, Co, and Si into the framework of AlPO_4_-5. Ion substitution in AlPO_4_-5 takes place through various mechanisms depending on the substituting element, with divalent and trivalent ions substituting Al, whereas tetravalent ions such as silicon substitutes predominantly P at low Si content, whereas at higher Si ratios a pair of Si ions substitutes adjacent Al and P ions (Gaber et al., 2020). In the present set of experiments, a water/Al_2_O_3_ molar ratio of 100:1 and a metal/Al_2_O_3_ ratio of 5:100 were used. The XRD patterns of the resulting MeAPOs (Figure 3A) are in accordance with the AFI structure possessing all the AFI peaks, including the characteristic ones at 2θ of approximately 20, 22, and 23° that correspond to the reflections from the (210) (002) and (211) crystallographic planes, respectively (Karanikolos et al., 2008; Stoeger et al., 2012; Basina et al., 2018). Furthermore, the materials exhibit high crystallinity as no broad peaks or shoulders and no evidence of secondary crystalline phases/impurities were noticed. A closer look at the XRD patterns reveals that some peaks of the ion substituted AlPOs are not perfectly aligned with those of the AlPO_4_-5. Indeed, Figure 3B shows the three characteristic AFI peaks extending between 19 and 23°, which are attributed to the crystallographic (210), (002) and (211) planes where minor shifts are evident. These shifts are attributed to the ion substitution and incorporation into the framework since heteroatoms with different ionic radii are inserted into the lattice substituting Al and/or P. The ionic radius of alumina is 0.53 Å with all substituting metals having larger ionic radii, i.e., Fe: 0.645 Å, Mg: 0.72 Å, and Co: 0.745 Å. Silica possesses an ionic radius is 0.4 Å and has the ability to substitute phosphorus, which has an ionic radius 0.38 Å (SM2 mechanism). Notably, Fe exhibits the biggest expansion along the *c*-dimension. These shifts confirm the successful substitution of the metals into the AlPO framework. Analogous results were reported in our previous work, where AlPO_4_-5 metal substitution resulted in change of lattice parameters (Gaber et al., 2020).

**Figure 4:**
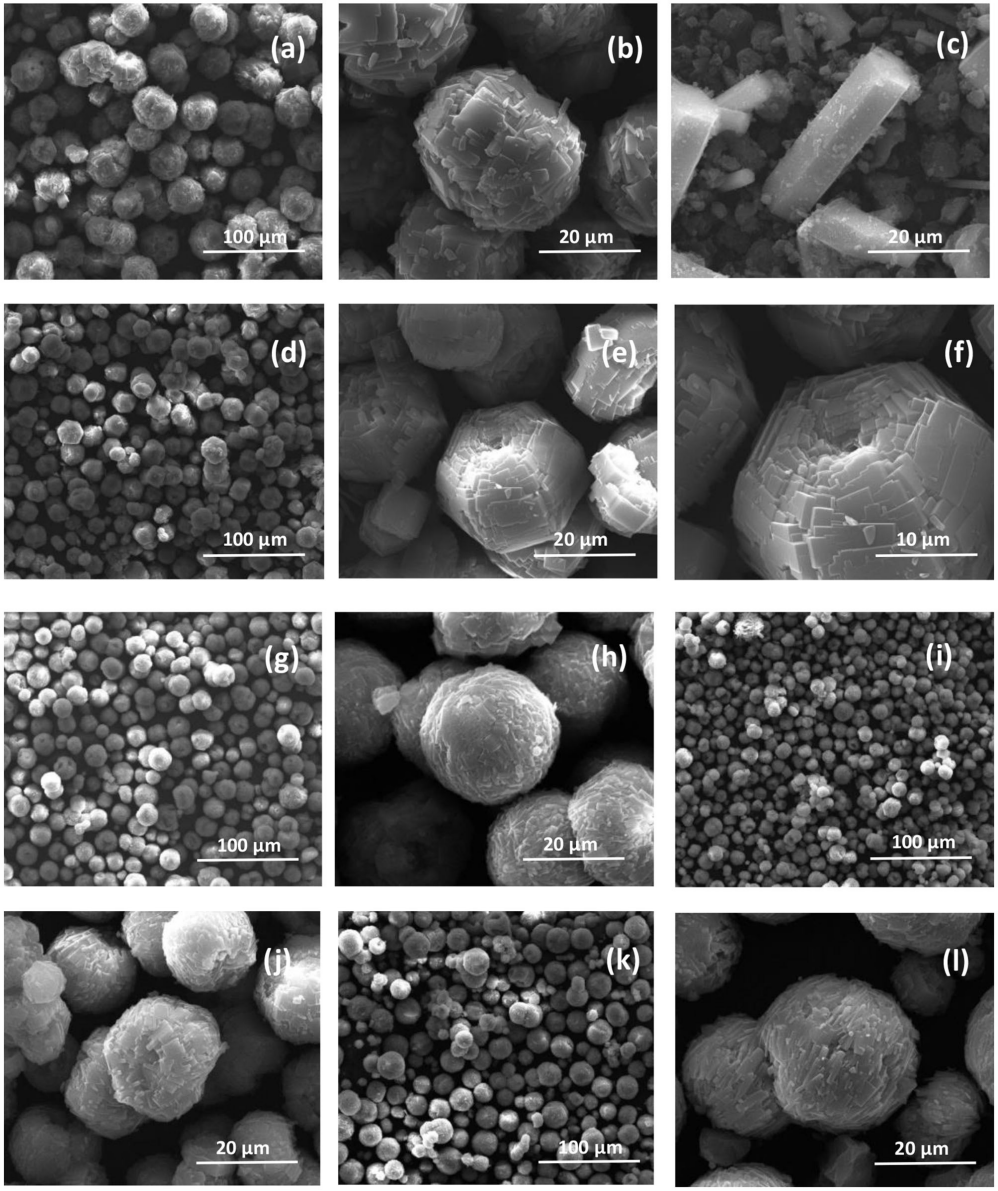
SEM images of **(a,b)** 100AlPO_4_-5, **(c)** 400AlPO_4_-5, **(d–f)** FeAPO-5, **(g,h)** MgAPO-5, **(i,j)** CoAPO-5, and **(k,l)** SAPO-5 adsorbents.

SEM images of calcined AlPO_4_-5 and MeAPO-5 are depicted in Figure 4. The materials are comprised of crystalline particles, thus confirming the XRD findings. All images correspond to adsorbents generated from dense reaction mixtures (H_2_O/Al_2_O_3_ molar ratio of 100), except Figure 4c that corresponds to dilute reaction mixture (H_2_O/Al_2_O_3_ molar ratio of 400) and the crystals exhibit columnar morphology. According to the low magnification images, the particles of the materials with H_2_O/Al_2_O_3_ molar ratio of 100 have spherical shape with uniform sizes that range between 20 and 30 μm. In high magnification, it is evident that the spherical particles are agglomerates of flat rectangular-like small crystals. In FeAPO-5 (Figures 4d,f), an analogous morphology is observed, whereas it is evident that the crystal aggregates tend to acquire a more hexagonal shape (Figure 4f) following the AFI crystal structure. This reveals that the particles in this adsorbent have been formed not by simple physical aggregation of the small individual crystallites, but rather a considerable degree of interaction and coalescence have taken place upon crystal growth.

The MgAPO-5 crystal aggregates (Figures 4g,h) exhibit spherical shape with an average particle diameter of 20 μm. The particle size of MgAPO-5 is smaller than that of FeAPO-5, whereas the aggregates seem to have a more perfect spherical shape compared to the hexagonal configuration observed in FeAPO-5. This reveals that interaction and coalescence between the individual small crystallites in MgAPO-5 is lower compared to that in FeAPO-5. CoAPO-5 (Figures 4i,j) also mainly consists of spherical particles, but they are quite heterogeneous since some of them have holes at their axis. The majority of SAPO-5 crystals are spherical, but some larger, irregularly shaped aggregates have also formed by further merging of the spherical particles, as shown in Figures 4k,l.

The morphology of AlPO_4_-5 prepared at high dilution rate (H_2_O/Al_2_O_3_ molar ratio in the synthesis mixture of 400, Figure 4c) reveals mainly columnar, hexagonal monocrystals (Cheung et al., 2012; Gaber et al., 2020). This is attributed to the fact that dilution of the reaction gel decreases the nucleation rate (Du et al., 1997), and favors preferential growth along the c-axis of the crystals. Indeed, the spheres formed in the samples using dense synthesis mixtures (H_2_O/Al_2_O_3_ molar ratio of 100) are comprised of smaller rectangular-like crystals that are tightly interconnected. The progress of crystallization controls the size of the spherical aggregates and prove to be rather homogeneous. FeAPO-5 particles tend to acquire a hexagonal shape in accordance to the AFI morphology compared to the rest of materials grown using dense reaction mixtures, thus indicating a closer interaction and coalescence among the individual flat-like crystallites that comprise each aggregate. This can be correlated to the XRD observations (Figure 3B), where FeAPO-5 was shown to exhibit the largest shift in the (002) peak, which may be also partially associated to the interface Fe ions shared among coalesced crystallites. Increasing the dilution slows down the crystallization rate, thus forming hexagonal, rod-like shape crystals following preferential growth along the c-axis (Iwasaki et al., 2003; Karanikolos et al., 2008). However, due to slow crystallization, it is possible that these samples may also possess some amorphous areas (Utchariyajit and Wongkasemjit, 2008) and low crystallinity regions.

**Figure 5:**
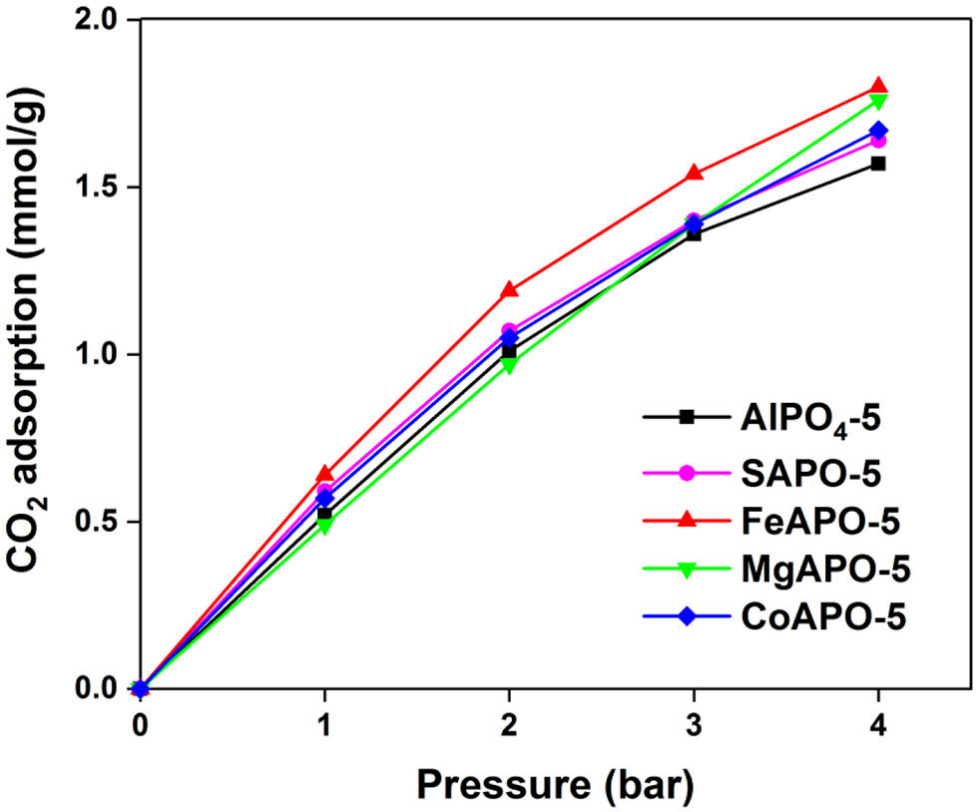
CO_2_ adsorption of various metal substituted AlPOs at 25°C.

The CO_2_ adsorption results of the metal substituted AlPOs and the parent AlPO_4_-5 are shown in Figure 5 (data tabulated in Supplementary Table 2). Overall, MeAPO-5 samples exhibit higher capacity than the parent AlPO_4_-5 indicating that metal substitution promotes CO_2_ adsorption. Among all MeAPO-5 samples, FeAPO-5 exhibits the highest capacity throughout the pressure range tested, reaching a maximum value of 1.8 mmol/g at 4 bar, which is 15% higher than that of the parent AlPO_4_-5 at the same conditions, owing to the substitution of Al^3+^ with Fe^3+^ in the lattice. At the lower pressure range of up to 3 bar, the Fe- and Co-substituted analogs exhibit higher capacity than the Mg-substituted one due to the fact that the presence of transition metal elements (Fe and Co) in the lattice tend to enhance the affinity between CO_2_ with the inorganic framework compared to alkali earth metals (Mg) and weak metals (Al) (Yu et al., 2018).

The CO_2_ capacity of MgAPO-5 is the lowest at low CO_2_ partial pressures even when compared to the parent AlPO_4_-5 adsorbent, whereas at pressures higher than 2.5 bar it displays an increase reaching at 4 bar at an almost same value as that of FeAPO-5. Strong ionic character and bond length in this case (Mg-O 1.969 Å) could influence CO_2_ sorption (Caskey et al., 2008; Yazaydin et al., 2009), with a possible clustering effect of CO_2_ molecules in the vicinity of Mg within the pores taking place at higher pressures. In addition, diffusion limitations within the pores as well as in the interstitial spaces among the crystallites in each particle agglomerate could be overcome as pressure increases. CoAPO-5 and SAPO-5 contain acidic sites within their framework due to charge imbalance upon ion substitution. Their sorption capacity is slightly higher compared to the parent AlPO_4_-5 adsorbent (by 6% and 4%, respectively). Analogous results were reported using *in situ* IR spectroscopy studies on SAPO-56 adsorbents possessing a high concentration of acid sites (Cheung et al., 2012). Effect of ion substitution on the lattice parameters may play also a role in CO_2_ adsorption. The substituting metals have two choices in AlPO_4_-5, i.e., to substitute Al and P positions. In general, Si will preferentially substitute for P, and divalent and trivalent ions will replace Al (Wilson et al., 1982). Substitution by transition metals with higher ionic radius (Fe, Mg, and Co) will generally increase the value of lattice parameters than low ionic radius elements such as Si (Kaneko and Rodríguez-Reinoso, 2019), thus enhancing the CO_2_ adsorption capacity.

### Effect of Metal Content

The enhanced CO_2_ adsorption capacity of FeAPO-5 compared to all other AlPO samples tested led us to further investigate this material by varying the molar composition of iron in the precursor mixture. An initial molar ratio of Fe/Al_2_O_3_ of 5:100 was used as a basis, and a set of materials with three more Fe/Al_2_O_3_ molar ratios, namely, 2.5:100, 7.5:100, and 10:100, were additionally synthesized and studied.

**Figure 6:**
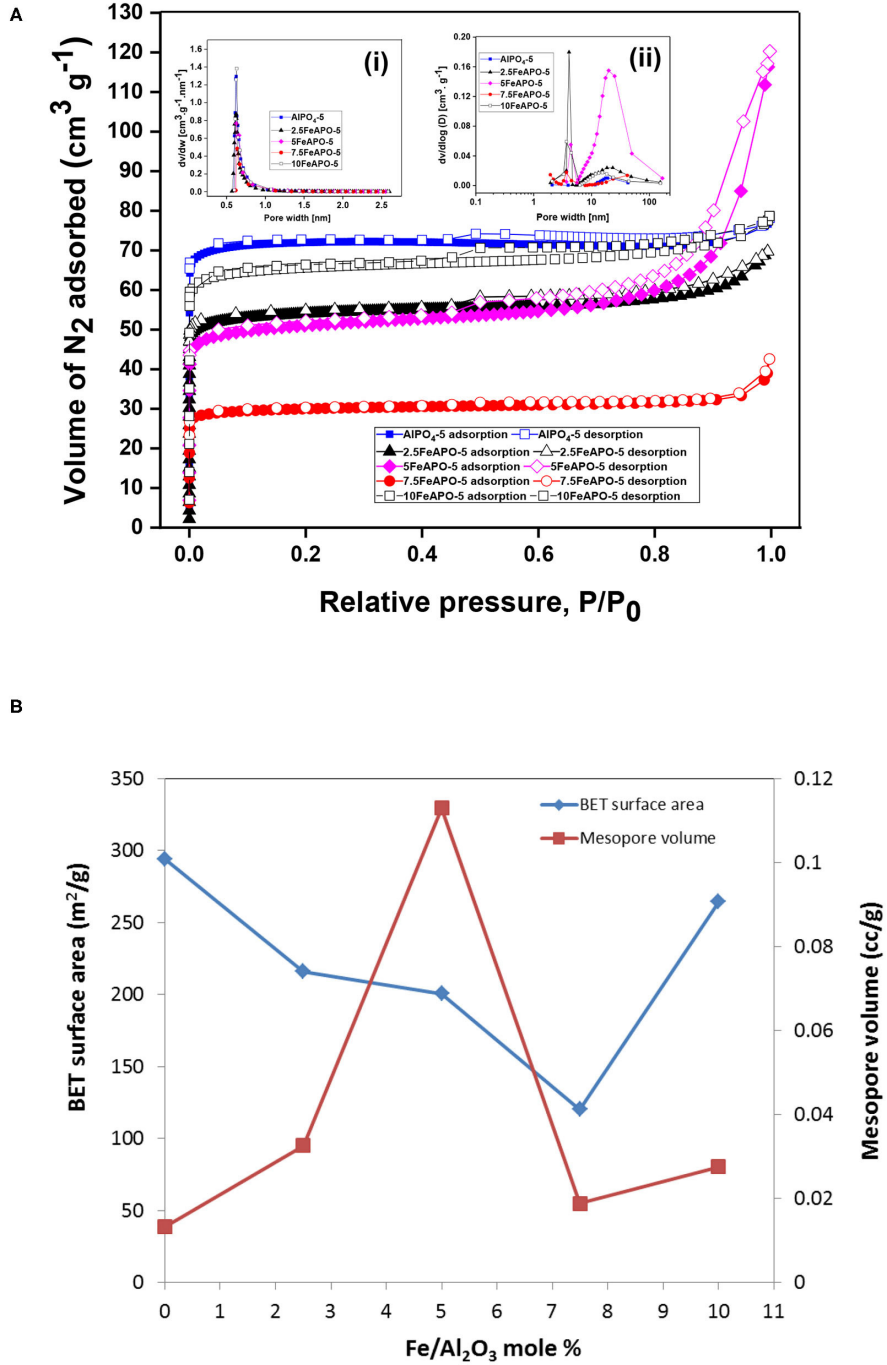
**(A)** Liquid N_2_ adsorption–desorption isotherms of FeAPOs. Inset (i): Micropore size distribution determined by the HK method. Inset (ii) BJH-derived mesopore size distribution. **(B)** BET surface area and mesopore volume as a function of Fe/Al_2_O_3_ molar content (with respect to %Al_2_O_3_) in the synthesis mixture.

The liquid N_2_ adsorption–desorption isotherms of the FeAPO-5 adsorbents are shown in Figure 6A, while the resulting physical and pore properties estimated from BET analysis, BJH, and HK methods are presented in Table 2. All adsorbents exhibit a steep nitrogen uptake at a low relative pressure (P/P_o_) confirming their microporous nature. The adsorbents display a type-IV isotherm with hysteresis loops extending across relative pressures of 0.45 to 0.9, which confirm also the existence of mesopores. Both micropore and mesopore size distributions are shown in insets i and ii, respectively. Among the tested samples, the steepest increase at high relative pressures (*P*/*P*_o_ > 0.8) and the most extensive hysteresis loop was observed for 5FeAPO-5, which are indicative of an extended mesoporous network (Figure 6B). According to the micropore size distribution, all-metal substituted samples possess a narrow peak centered at ~0.7 nm, which confirms their AFI structure (Karanikolos et al., 2008). From Table 2, the BET surface area of FeAPO-5 adsorbents varies from 120 to 264 m^2^/g, the pore volume from 0.06 to 0.18 cc/g, and the average pore diameter from 1.8 to 3.5 nm. The relatively wide ranges observed reveal the considerable affect that the metal incorporation induces to the physical properties of the adsorbents.

**Table 2:** Physical properties of FeAPO-5.

**Adsorbent**	**BET surface**	**Average pore**	**Total pore**	**Micropore**	**Mesopore**
	**area**	**diameter**	**volume**	**volume**	**volume**
	**(m^**2**^/g)**	**(nm)**	**(cc/g)**	**(cc/g)**	**(cc/g)**
AlPO_4_-5	294.1	1.60	0.1196	0.1063	0.0133
2.5FeAPO-5	215.8	1.98	0.1075	0.0749	0.0326
5FeAPO-5	200.6	3.47	0.1797	0.0668	0.1129
7.5FeAPO-5	120.4	1.93	0.0610	0.0421	0.0189
10FeAPO-5	264.4	1.80	0.1206	0.0930	0.0276

**Figure 7:**
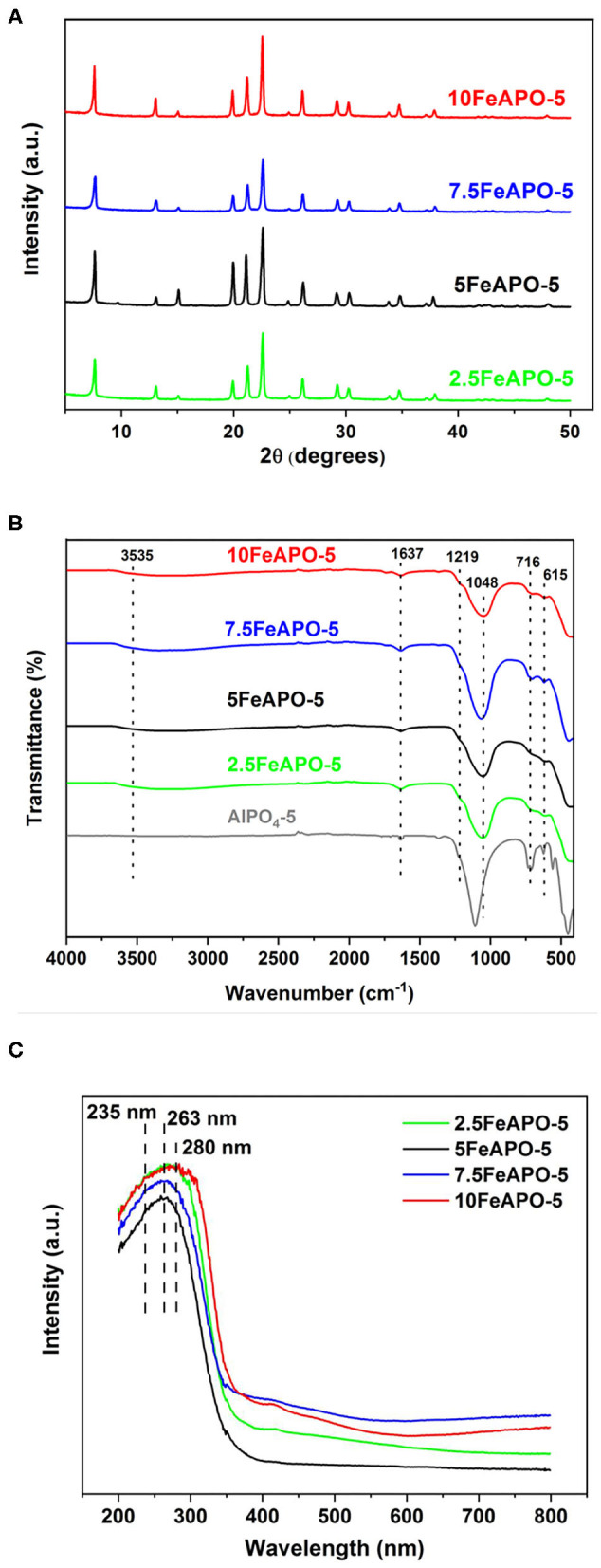
**(A)** XRD, **(B)** FTIR, and **(C)** UV-Vis DRS analysis of FeAPO-5 adsorbents.

To shed more light on the structural effects of the metal content, we also performed XRD, FTIR, and UV-Vis DRS analysis of the FeAPO-5 adsorbents. According to the XRD patterns (Figure 7A), the structural integrity of the AFI framework is retained for the whole range of Fe/Al_2_O_3_ % molar ratios tested (2.5–10), with no evidence of metal incorporation effects in the AFI crystallinity or appearance of secondary phases/impurities. FTIR spectra are shown in Figure 7B. The band at 3,535 cm^−1^ is ascribed to –OH functional groups, whereas the bands at 1,219, 716, and 615 cm^−1^ are ascribed to the asymmetric and symmetric stretching vibrations of the Al-O-P units (Zhang et al., 2020). Comparing the FeAPO-5 samples with the non-substituted AlPO_4_-5, the band at 562 cm^−1^ due to Al-O or P-O bending modes of AlPO_4_-5 framework is significantly suppressed upon Fe incorporation, whereas the band at 1,112 cm^−1^, which corresponds to stretching vibration of Al–O in combination with P–O (Chen and Jehng, 2003), is shifted to lower wavenumber (cm^−1^) values. Comparison among the various FeAPO-5 samples does not reveal any noticeable differences in FTIR bands as to differentiate among the Fe loadings and/or possible Fe segregation. UV-Vis DRS spectra of the Fe-substituted adsorbents are shown in Figure 7C. A dominant peak centered at 263 nm is evident for all FeAPO-5 samples with a shoulder at 235 nm attributed to the ligand to metal charge transfer of Fe^3+^ in [FeO_4_]^−^ tetrahedral geometry (Mohapatra et al., 2002). The intensity of the above band for the unsubstituted AlPO_4_-5 is negligible compared to the iron containing samples (Feng et al., 2016). Notably, for the sample with the highest metal content (10FeAPO-5) the above peak is shifted to higher wavelengths, which is indicative of increased amount of octahedral complexes in extra-framework positions, and the distribution state for the Fe species corresponding to 280 nm in particular can be isolated or clustered (Feng et al., 2016). The broad band between 400 and 500 nm is due to Fe d–d transitions and is also indicative of clustering of iron species (Wei et al., 2008). Notably, the intensity of the above band for the 5FeAPO-5 sample is minimal.

**Figure 8:**
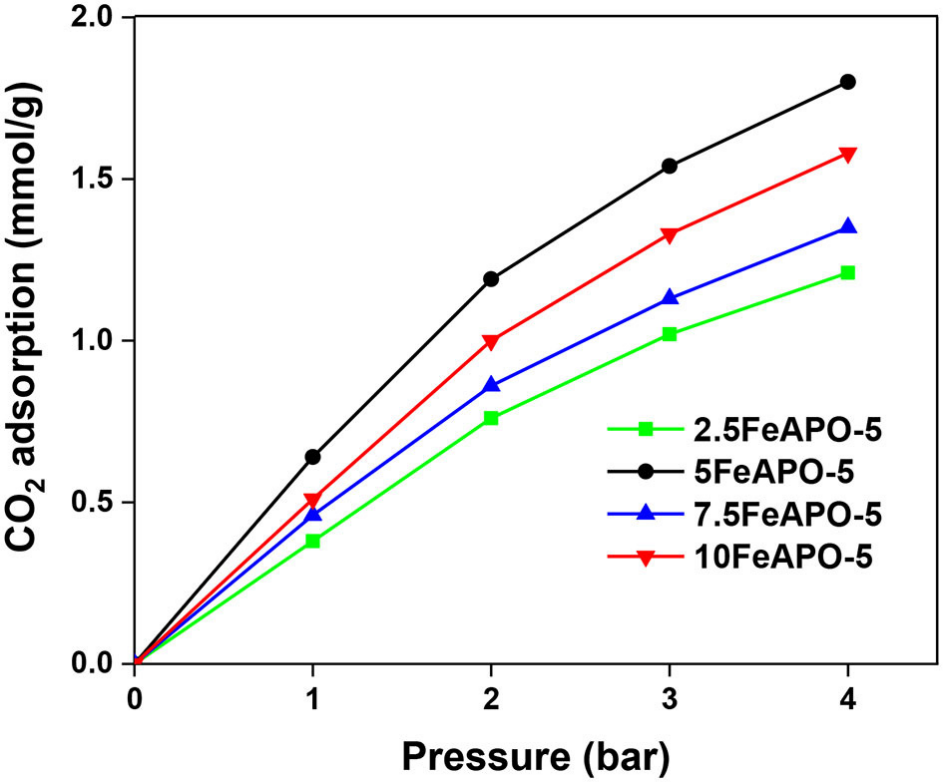
CO_2_ adsorption of FeAPO-5 adsorbents with different metal content at 25°C.

The FeAPO-5 adsorbents exhibit both micropores with an average diameter of ~0.7 nm, which is bigger than the kinetic diameter of CO_2_ (0.33 nm), thus posing no kinetic restriction for CO_2_ adsorption, as well as a relatively extended mesoporous network (Figure 6A). In such configuration, at low partial pressure of CO_2_, monolayer adsorption and adsorption on the micropores are anticipated to occur first, whereas mesopores are filled as pressure increases and adsorbate–adsorbate interactions are being enhanced. The CO_2_ adsorption capacity of the FeAPO-5 adsorbents with different metal concentrations are shown in Figure 8 (data also in Supplementary Table 3). According to Figure 6B, the BET surface area decreases with Fe content until a Fe/Al molar ratio of 7.5:100 and then it increases again. The mesopore volume follows an opposite trend, i.e., it increases with Fe content, exhibits a maximum at Fe/Al ratio of 5:100, and then it decreases. As shown by UV-Vis DRS analysis above, for high metal concentration in the synthesis mixture, a portion of the available Fe forms clusters, and it is not incorporated into the AFI framework as tetrahedrally coordinated ions by substituting Al^3+^. The existence of amorphous Fe^3+^ phase for high metal loading has been confirmed also before via various spectroscopic and chemical probe methods (Das et al., 1992). Consequently, the CO_2_ adsorption capacity does not exhibit a regular trend as Fe content increases, rather it exhibits a maximum for the sample corresponding to Fe/Al_2_O_3_ ratio of 5:100, which exhibits the higher volume of mesopores and the minimum portion of extra-framework or clustered iron as revealed by the UV-Vis analysis. Indeed, the adsorption capacity of 5FeAPO-5 at low pressure (1 bar) is 0.64 mmol/g and at high pressure (4 bar) it becomes 1.8 mmol/g, corresponding to an incremental increase of 1.16 mmol/g from the above low to high pressure values, which is the highest increment among the FeAPO-5 adsorbents tested. This is attributed to the contribution of mesopores in the adsorption capacity which becomes dominant at higher pressures. Conclusively, Fe incorporation enhances the mesoporosity despite the fact that it decreases the surface area. The latter is more important for relatively low metal content causing a reduction in CO_2_ capacity compared to pure AlPO_4_-5, yet as metal content increases, the mesoporosity formation becomes dominant and brings the capacity to higher values than that of the pure AlPO_4_-5 at same conditions. However, for high metal loadings, some Fe prefers to cluster upon growth forming amorphous Fe^3+^ phases and/or extra-framework species thus associated to reduced mesoporosity and lower CO_2_ capacity.

### Effect of Reaction Mixture Dilution and Associated AlPO_4_-5 Morphology

**Figure 9:**
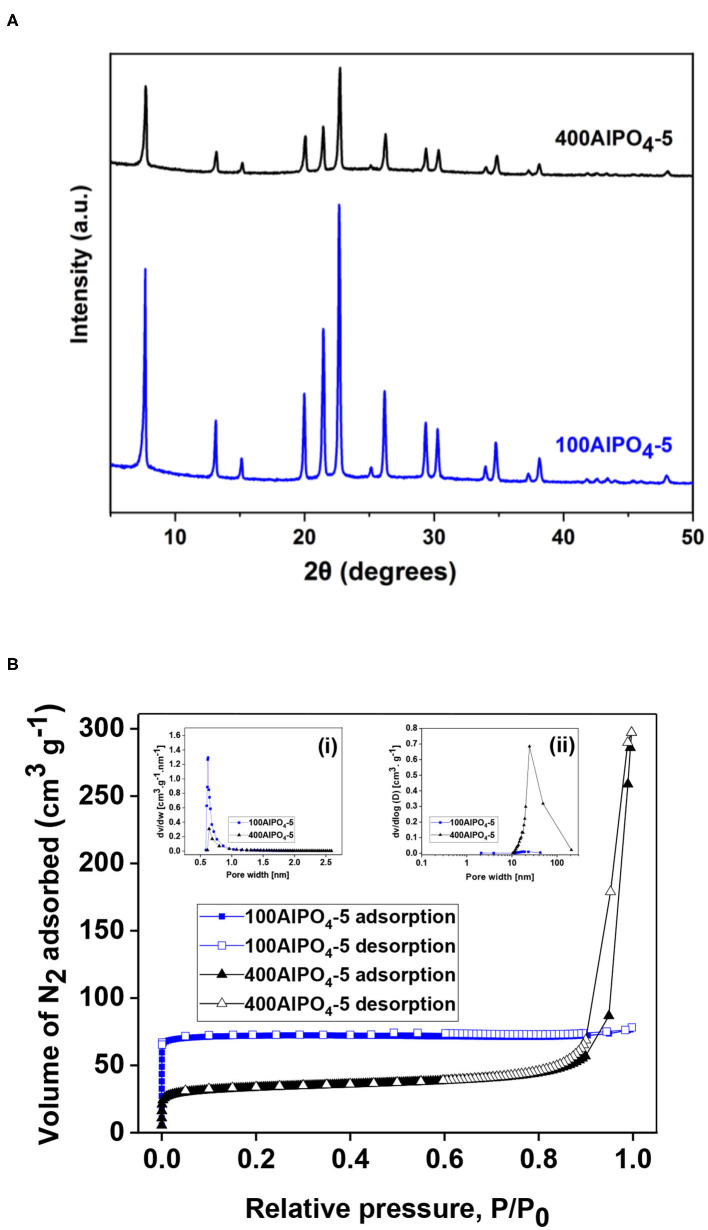
**(A)** XRD patterns for AlPO_4_-5 crystals corresponding to two different dilution rates in the reaction mixture, i.e., H_2_O/Al_2_O_3_ molar ratio of 100 and 400 (100AlPO_4_-5 and 400AlPO_4_-5). **(B)** N_2_ adsorption isotherms of 100AlPO_4_-5 and 400AlPO_4_-5 at 77K, inset (i): micropore size distribution determined by the HK method, inset (ii): BJH mesopore size distribution.

In our previous work we have shown that a critical parameter that strongly affects crystal morphology upon AlPO_4_-5 growth is the water content in the synthesis mixture (Karanikolos et al., 2008). Specifically, it was demonstrated that dense reaction mixtures favor growth perpendicular to the AFI channels, i.e., along the a-b directions, while diluted reaction mixtures induce preferential growth along the c-direction, i.e., parallel to the channels. Accordingly, we employed here two different dilutions in the reaction mixture, namely, H_2_O/Al_2_O_3_ molar ratio of 100 (sample 100AlPO_4_-5) and 400 (sample 400AlPO_4_-5), which resulted in spherical agglomerates of small flake-like crystallites, and columnar monocrystals, respectively (Figures 4A–C). From XRD analysis (Figure 9A), it is evident that both samples possess the AFI structure, yet the lower-dilution sample displays a higher crystallinity when compared to the high-dilution one.

The liquid N_2_ adsorption isotherms and pore size distributions of the 100AlPO_4_-5 and 400AlPO_4_-5 adsorbents are shown in Figure 9B, and the associated physical and porosity properties are presented in Table 3. It is evident that diluted reaction mixtures yield AlPO_4_-5 with lower surface area and micropore volume, yet enhanced mesoporosity. The shape of the isotherms also confirms this observation, as the isotherm of 100AlPO_4_-5 approaches that of type-I revealing a strongly microporous nature with negligible contribution from larger pores, whereas that corresponding to 400AlPO_4_-5 is a type-IV isotherm with a steep rise in adsorption at *P*/*P*_0_ > 0.85 and an enhanced hysteresis loop revealing capillary condensation in mesopores. Indeed, the surface area drops from 294 to 125 m^2^/g while the mesopore volume increases from 0.0133 to 0.4153 cc/g for the 100AlPO_4_-5 and 400AlPO_4_-5 adsorbents, respectively. The micropore size distribution (Figure 9B, inset ii) is narrow averaging at ~ 0.7 nm for both materials, in accordance to AFI structure, whereas the portion of larger pores is relatively low for 100AlPO_4_-5, and becomes significant for 400AlPO_4_-5, for which an average mesopore width of 25 nm is evidenced from Figure 9B, inset.

**Table 3:** Physical properties of 100AlPO_4_-5 and 400AlPO_4_-5.

**Adsorbent**	**BET surface**	**Average pore**	**Total pore**	**Micropore**	**Mesopore**
	**area**	**diameter**	**volume**	**volume**	**volume**
	**(m^**2**^/g)**	**(nm)**	**(cc/g)**	**(cc/g)**	**(cc/g)**
100AlPO_4_-5	294.1	1.60	0.1196	0.1063	0.0133
400AlPO_4_-5	125.2	13.0	0.4509	0.0356	0.4153

**Figure 10:**
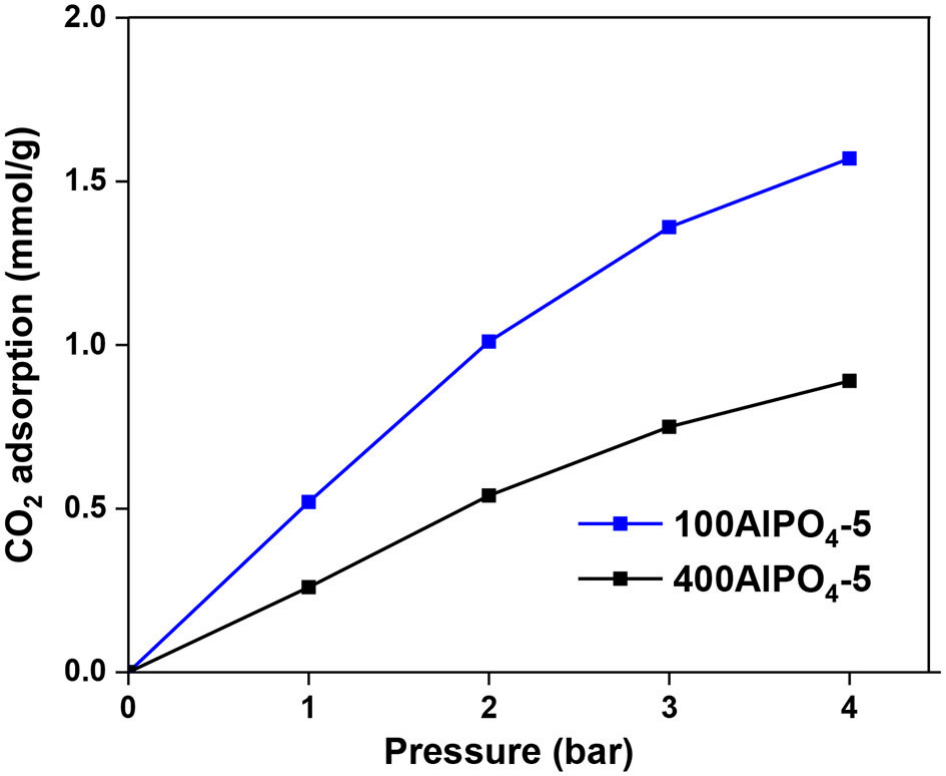
CO_2_ adsorption of AlPO_4_-5 adsorbents corresponding to two different reaction mixture dilution rates and associated crystal morphologies at 25°C.

The CO_2_ adsorption isotherms and associated capacities for the AlPO_4_-5 adsorbents corresponding to low and high dilutions of the reaction mixture are shown in Figure 10. It is evident that dense reaction mixtures (100AlPO_4_-5) yield adsorbents that exhibit higher capacity compared to diluted ones (400AlPO_4_-5). Indeed, 100AlPO_4_-5 exhibits an adsorption capacity of 1.57 mmol CO_2_/g at 4 bar and 25°C, which is almost double than that of 400AlPO_4_-5. As discussed earlier for the Fe-substituted adsorbents, a high mesopore volume is favorable for enhancing CO_2_ capacity. Nevertheless, this does not seem to be the case for the non-substituted materials where substituting heteroatoms do not exist. Here, the effect of BET surface area reduction for the adsorbent corresponding to high dilution rate of the reaction mixture is more dominant than the mesoporosity formation, thus causing a decrease in CO_2_ capacity compared to the adsorbent originated from dense mixture. In addition, a high average mesopore diameter (25 nm in the case of 400AlPO_4_-5) might decrease the interaction potential, affinity between CO_2_-CO_2_, and retention upon multilayer adsorption.

### Isosteric Heat of Adsorption

**Figure 11:**
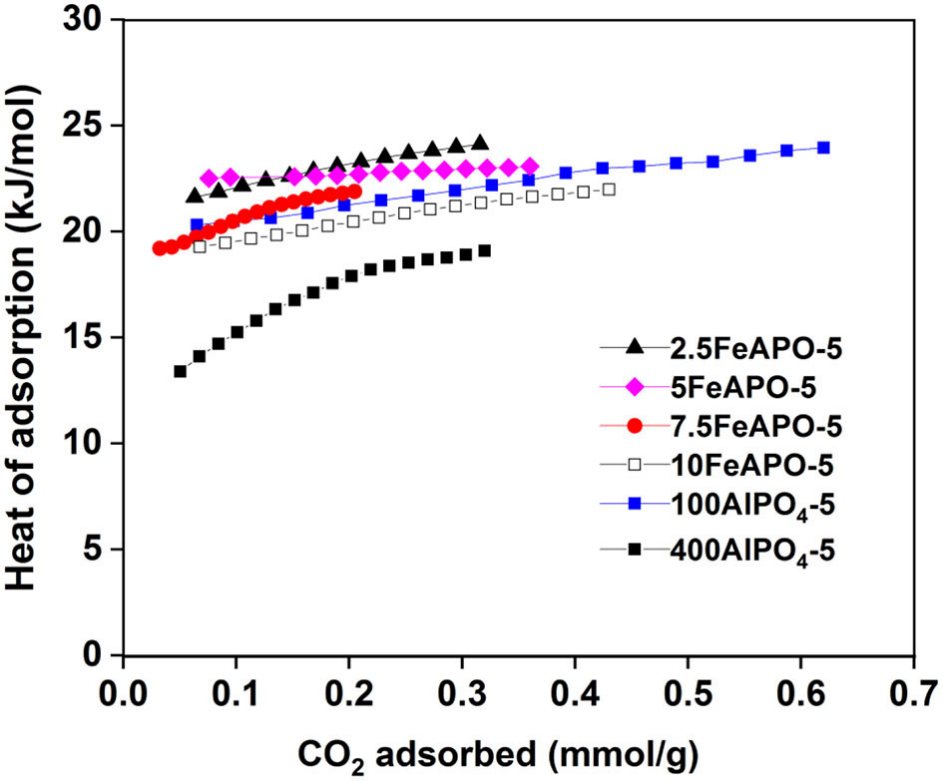
Isosteric heat of adsorption of different AlPO_4_-5 and FeAPO-5 adsorbents.

The isosteric heat of adsorption concerns the amount of energy that is released when CO_2_ adsorbs onto the adsorbent surface at a fixed coverage. Consequently, at least the same amount of energy must be added in order for the CO_2_ to be desorbed from the adsorbent upon regeneration. In addition, the isosteric heat of adsorption values provide a quantifiable indicator of the affinity of an adsorbate molecule toward the adsorbent surface. CO_2_ adsorption isotherms were collected at three different temperatures in order to calculate the heat of adsorption of the AlPO adsorbents at different CO_2_ loadings. The isosteric heat of adsorption values were determined based on the Clausius–Clapeyron equation using the obtained isotherm data, and the results are depicted in Figure 11.

The heat of adsorption values for all the studied adsorbents are lower than 25 kJ/mol throughout the tested coverages, which indicates that the mechanism of CO_2_ adsorption in these materials is physisorption, in line to the reported literature (Simmons et al., 2011). As such, they hold a high potential for PSA-based capture application as desorption can easily be enabled by pressure swing without the need of thermal regeneration. According to the obtained profiles, the heat of adsorption increases as the CO_2_ loading increases for all the studied adsorbents, implying that, as coverage increases, adsorbate–adsorbate (CO_2_-CO_2_) interactions are stronger than adsorbate–adsorbent interactions on CO_2_-philic surface sites. Concerning the Fe-substituted analogs in particular, the ones corresponding to relatively low Fe/Al_2_O_3_ molar ratio in the synthesis mixture (2.5:100 and 5:100) exhibit the highest heat of adsorption values compared to the rest of tested adsorbents. This indicates that the incorporated metal enhances the physical binding, a fact that is more pronounced for 5FeAPO-5 at low coverage indicating a stronger CO_2_ interaction on the adsorbent surface upon monolayer formation. Notably, the above mentioned adsorbent exhibited the highest uptake compared to the rest of the ion-substituted materials tested as well as to the pure AlPO_4_-5 (Figures 5, 8). Furthermore, the heat of adsorption curve of 5FeAPO-5 is almost flat, which is attributed to high crystallinity and homogenous surface with a uniform distribution of the Fe heteroatoms into the framework lattice that enhance binding. Notably, this adsorbent contains the lowest portion of extra-framework or clustered iron among all FeAPO-5 materials tested (Figure 7C). As Fe content in the synthesis mixture increases the heat of adsorption, and thus the CO_2_ binding strength with the adsorbent surface decreases, a result that is in agreement to the observed reduced CO_2_ adsorption capacity for 7.5FeAPO-5 and 10FeAPO-5, which is attributed to the tendency of the readily available Fe ions at these high metal concentrations to cluster in the synthesis mixture and yield formation of amorphous Fe^3+^ phases upon crystal growth (Das et al., 1992), as also discussed above (Figure 7C), and/or remain as extra-framework species partially blocking active surface sites.

The low value of heat of adsorption for the 400AlPO_4_-5 adsorbent, which corresponds to diluted reaction mixture, indicates that the morphology and textural and surface properties of this material, including the large pore width, large mesopore volume, and rod-like crystal morphology, promote a very weak binding with the surface, which is in agreement to the low CO_2_ adsorption capacity obtained for this material. In addition, the heat of adsorption curve as a function of coverage for this adsorbent exhibits the largest slope among all tested adsorbents for the low coverage range (up to 0.22 mm/g). This is indicative of the initially weak interaction of CO_2_ with the surface and during monolayer adsorption, which becomes stronger as coverage increases due to more dominant adsorbate–adsorbate interactions. At higher coverages (>0.22 mmol/g), the slope becomes lower and almost equalizes with that of the other tested adsorbents.

### CO_2_ Adsorption Kinetics

Understanding of the adsorption kinetics of an adsorbent is vital in designing PSA systems (Loganathan et al., 2014). Various adsorption kinetic models have been employed, among which, the pseudo–first-order kinetics or Lagergren model (Liu et al., 2014) is widely used for CO_2_ adsorption. The pseudo–first-order equation models a reversible interaction between adsorbent and adsorbate. This is suitable for describing the physical adsorption of CO_2_ on solid adsorbents (Serna-Guerrero and Sayari, 2010; Loganathan et al., 2014). The pseudo–first-order model is governed by Equation (1):

(1) $$\begin{array}{r} \frac{dq_{t}}{dt}=k\left(q_{e}-q_{t}\right) \end{array}$$

where *q*_e_ and *q*_t_ (mg/g) represent the amount of CO_2_ adsorbed at e*q*uilibrium and at a given time “*t*”, respectively, and k (min^−1^) is the first order rate constant. The relevant boundary conditions are BC1: *t* = 0, *q*_t_ = 0 and BC2: *t* = ∞, *q*_t_ = *q*_e_. Applying the above boundary conditions to Equation (1) while solving the aforementioned differential equation leads to Equation (2).

(2) $$\begin{array}{r} \log\left(q_{e}-q_{t}\right)=\log\left(q_{e}\right)-\left(\frac{k}{2.303}\right)t \end{array}$$

**Figure 12:**
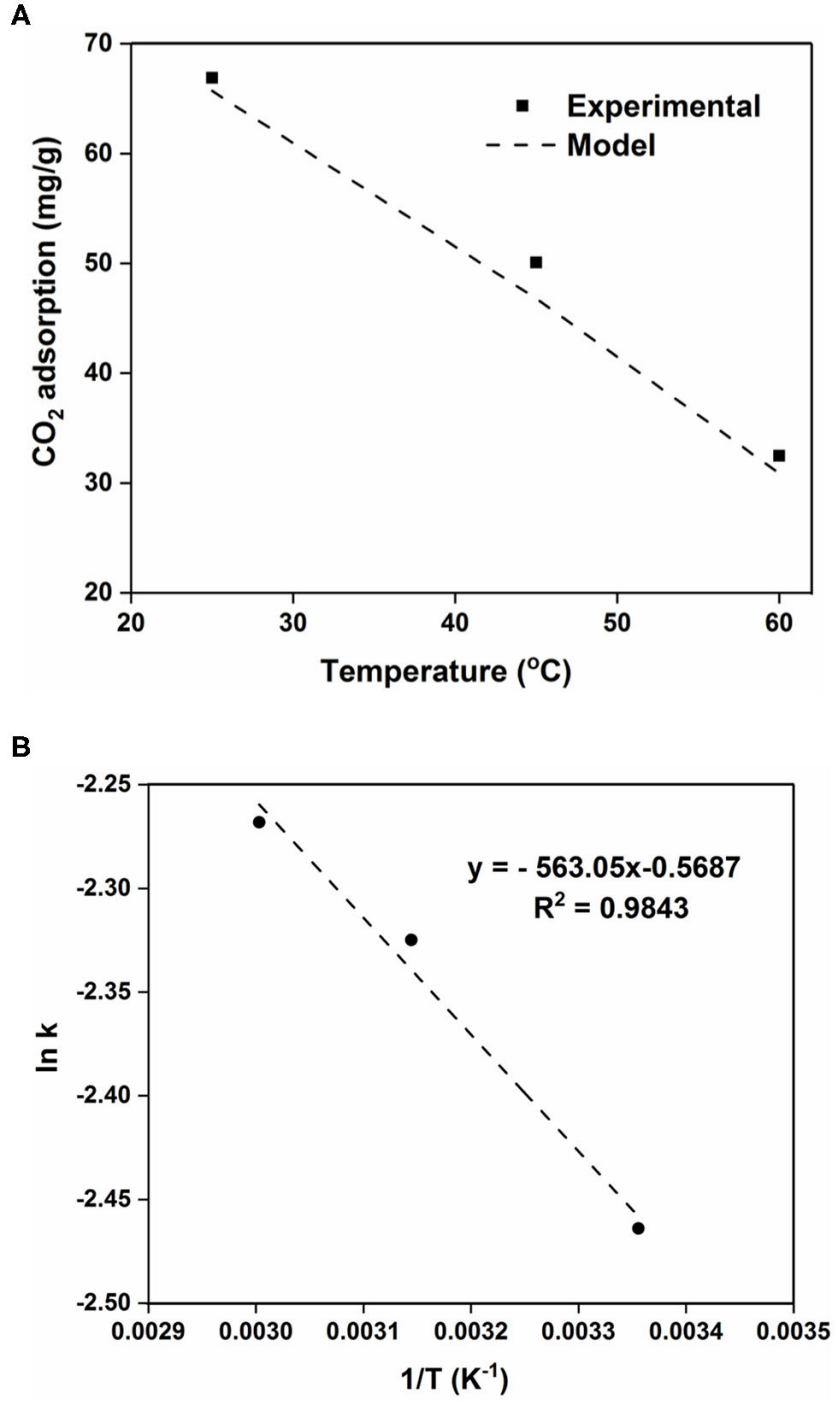
**(A)** Comparison of experimental data vs. model fitting data. **(B)** Arrhenius plot for the kinetic rate constant obtained by the linear Lagergren model for the 100AlPO_4_-5 adsorbent.

The higher CO_2_ adsorption capacity and rate of the 100AlPO_4_-5 adsorbent compared to the 400AlPO_4_-5 analog, as confirmed by both equilibrium data at various pressures (Figure 10), and kinetic data up to 4 bar (Supplementary Figure 3) led us to further investigate the kinetic behavior of this adsorbent by conducting adsorption experiments at three different temperatures, i.e., 25, 45, and 60°C up to a pressure of 4 bar (Supplementary Table 5 and Supplementary Figure 4). The CO_2_ adsorption capacity decreases as temperature increases confirming the physisorption mechanism. In order to calculate the adsorption rate constant k for the three different temperatures, the adsorption data at the different temperatures were used to plot log (*q*_e_ – *q*_t_) vs. t/2.303 (Supplementary Figure 5). The resulting linear fit slope is the adsorption rate constant (k), and the *y*-intercept is log *q*_e_ (Liu and Shen, 2008; Liu et al., 2014). The adsorption rate constant increases as temperature increases (Table 4) yet with relatively slight differences indicating that the adsorption of CO_2_ on AlPO_4_-5 is rather thermodynamically limited. The equilibrium adsorption capacity (q_e_) was also calculated from the pseudo–first-order model and compared with the experimentally measured one (Supplementary Table 5). The model predicted the equilibrium adsorption capacity with an average relative error of 1.8–6.5%, and the value of the correlation coefficient (*R*^2^) of the model is around 0.98. Thus, the pseudo–first-order model proves to be qualitatively and quantitatively suitable for modeling of CO_2_ adsorption kinetics of the studied AlPO_4_-5 adsorbents as shown Figure 12A.

**Table 4:** Adsorption rate constant (k) at different temperatures and up to 4 bar for 100AlPO_4_-5.

**Temperature (°C)**	**Constant rate k (min^**−1**^)**
25	0.0851
45	0.0978
60	0.1035

The temperature dependence of rate constant (k) can be described by the Arrhenius equation (Equation 3), where *A* is the pre-exponential factor, *E* is the activation energy, *R* is the universal gas constant, and *T* is the temperature in absolute units.

(3) $$\begin{array}{r} k=Ae^{-\left(\frac{E}{RT}\right)} \end{array}$$

The plot of ln(k) vs. 1/*T* (Figure 12B) exhibits a linear profile, as anticipated from the linearized form of the Arrhenius equation. From the obtained slope, the activation energy for the CO_2_ adsorption on 100AlPO_4_-5 at 4 bar was calculated to be 4.7 kJ/mol. The obtained activation energy value is in close agreement to the reported values of CO_2_ adsorption on activated carbon and Zeolite 13X, where activation energies of 3.9 and 4.8 kJ/mol, respectively, were estimated up to CO_2_ pressure of 3 bar (Zhang et al., 2010).

## Conclusions

Effects of metal substitution, synthesis mixture composition and associated morphology manipulation, and activation procedure on AlPO_4_-5 were studied for CO_2_ adsorption. Activation by calcination in air at high temperature was found to completely open up the pores resulting in higher CO_2_ capacity compared to calcination at lower temperatures. Yet, upon activation by pyrolysis in inert atmosphere, low temperature (240°C) treatment enhanced CO_2_ interaction with the surface at low pressures (up to 1 bar) due to the existence of remnant carbon species in the pores from the partial decomposition of the SDA. Ion substitution by Fe, Mg, Co, and Si induced changes in lattice parameters and morphology, whereas the Fe-substituted adsorbents exhibited the highest capacity compared to the rest of metal-substituted analogs. Parametric variation of the Fe content in the synthesis mixture revealed that, at relatively low metal concentrations, mesopore volume increases and microporosity and BET surface area decrease with metal content. At these conditions, the strong interaction of CO_2_ with the framework Fe and the adsorbate–adsorbate interactions in the formed mesopores increased the CO_2_ capacity. For high metal concentrations, some Fe prefers to cluster upon growth forming amorphous Fe^3+^ phases and/or extra-framework species, thus resulting in reduced mesoporosity and lower CO_2_ capacity. Dense reaction mixtures yielded AlPO_4_-5 consisting of spherical agglomerates of 2D-like crystallites that were almost exclusively microporous in nature, whereas diluted reaction mixtures resulted in crystals of columnar morphology exhibiting significant mesoporosity. In contrast to the metal-substituted analogs and thus in the absence of metal-CO_2_ interactions, the microporosity and high surface area of the AlPO_4_-5 adsorbents corresponding to dense reaction mixtures were dominant factors compared to the enhanced mesoporosity of the ones grown from diluted mixtures, thus yielding higher CO_2_ capacity for the former materials.

The isosteric heat of adsorption values for all the studied adsorbents were lower than 25 kJ/mol indicating that the mechanism of CO_2_ adsorption is physisorption, which is suitable for PSA application. The Fe-substituted analog corresponding to a Fe/Al_2_O_3_ molar ratio in the reaction mixture of 5:100 exhibited the highest heat of adsorption at low coverage, indicating affinity and stronger physical binding of CO_2_ with the framework-incorporated Fe. Notably, this adsorbent exhibited the lowest portion of extra-framework or clustered iron. Kinetic analysis revealed that a pseudo–first-order model could describe well the CO_2_ adsorption kinetics of the AlPO_4_-5 adsorbents, with an activation energy of 4.7 kJ/mol at 4 bar and adsorption rate constants increasing with temperature, yet with rather slight differences. AlPO_4_-5–based adsorbents, though they exhibit rather moderate capacities, they are robust, thermally and chemically stable materials, they exhibit low heat of adsorption, thus potential for PSA application, and limited hydrophilicity compared to classical zeolites that makes them important for CO_2_ capture from wet streams. The parametric investigation performed in this work sheds light on main optimization factors and paves the way for further studies toward implementation at industrial scale.

## Data Availability Statement

All datasets generated for this study are included in the article/Supplementary Material.

## Author Contributions

AP performed the synthesis/growth, characterization of the adsorbents, and drafted part of the manuscript. KR performed the CO_2_ evaluation and drafted part of the manuscript. DK edited the manuscript and contributed in the supervision of AP. DR edited the manuscript and contributed in the supervision of the activities. YA contributed in the supervision of the activities, provided feedback and ideas, contributed in the design of the experiments, and edited the manuscript. GK set up and designed the project, attracted the funding, supervised the activities, and drafted/edited the manuscript. All authors contributed to the article and approved the submitted version.

## Conflict of Interest

The authors declare that the research was conducted in the absence of any commercial or financial relationships that could be construed as a potential conflict of interest.
