# Supplementary material for: Morphology, Activation, and Metal Substitution Effects of AlPO4-5 for CO2 Pressure Swing Adsorption
Source: Front Chem. 2020 Oct 6;8:568669. doi: 10.3389/fchem.2020.568669 (PMC7573157; doi:10.3389/fchem.2020.568669)
Supplement: Supplementary file 1 [file Table_1.doc]

# **Morphology, Activation and Metal Substitution Effects of AlPO_4_-5 for CO_2_ Pressure Swing Adsorption**

**Supplementary data**

**Supporting Tables:**

**Table S1:** CO_2_ adsorption capacity data of calcined and pyrolyzed AlPO_4_-5 samples at various pressures and at 25 °C.

| **Sample** | **Pressure (bar)** | **CO_2_ adsorption capacity (mmol CO_2_/g)** |
| --- | --- | --- |
| 400AlPO_4_-5.C | 0 | 0 |
|  | 1 | 0.41 |
|  | 2 | 0.83 |
|  | 3 | 1.13 |
|  | 4 | 1.33 |
| 700AlPO_4_-5.C | 0 | 0 |
|  | 1 | 0.48 |
|  | 2 | 0.95 |
|  | 3 | 1.28 |
|  | 4 | 1.53 |
| 240AlPO_4_-5.P | 0 | 0 |
|  | 1 | 0.43 |
|  | 2 | 0.8 |
|  | 3 | 1.11 |
|  | 4 | 1.32 |
| 400AlPO_4_-5.P | 0 | 0 |
|  | 1 | 0.36 |
|  | 2 | 0.82 |
|  | 3 | 1.15 |
|  | 4 | 1.38 |
| 700AlPO_4_-5.P | 0 | 0 |
|  | 1 | 0.47 |
|  | 2 | 0.92 |
|  | 3 | 1.24 |
|  | 4 | 1.48 |

**Table S2:** CO_2_ adsorption capacity data of AlPO_4_-5 and MeAPO-5 samples at various pressures and at 25 °C.

| **Sample** | **Pressure (bar)** | **CO_2_ adsorption capacity (mmol CO_2_/g)** |
| --- | --- | --- |
| AlPO_4_-5 | 0 | 0 |
|  | 1 | 0.52 |
|  | 2 | 1.01 |
|  | 3 | 1.36 |
|  | 4 | 1.57 |
| SAPO-5 | 0 | 0 |
|  | 1 | 0.59 |
|  | 2 | 1.07 |
|  | 3 | 1.40 |
|  | 4 | 1.64 |
| FeAPO-5 | 0 | 0 |
|  | 1 | 0.64 |
|  | 2 | 1.19 |
|  | 3 | 1.54 |
|  | 4 | 1.80 |
| MgAPO-5 | 0 | 0 |
|  | 1 | 0.49 |
|  | 2 | 0.97 |
|  | 3 | 1.39 |
|  | 4 | 1.76 |
| CoAPO-5 | 0 | 0 |
|  | 1 | 0.57 |
|  | 2 | 1.05 |
|  | 3 | 1.39 |
|  | 4 | 1.67 |

**Table S3**: Data for CO_2_ adsorption of FeAPO-5 with different metal content at various pressures and at 25 °C.

| **Sample** | **Pressure (bar)** | **CO_2_ adsorption capacity (mmol CO_2_/g)** |
| --- | --- | --- |
| 2.5FeAPO-5 | 0 | 0 |
|  | 1 | 0.38 |
|  | 2 | 0.76 |
|  | 3 | 1.02 |
|  | 4 | 1.21 |
| 5FeAPO-5 | 0 | 0 |
|  | 1 | 0.64 |
|  | 2 | 1.19 |
|  | 3 | 1.54 |
|  | 4 | 1.80 |
| 7.5FeAPO-5 | 0 | 0 |
|  | 1 | 0.46 |
|  | 2 | 0.86 |
|  | 3 | 1.13 |
|  | 4 | 1.35 |
| 10FeAPO-5 | 0 | 0 |
|  | 1 | 0.51 |
|  | 2 | 1.00 |
|  | 3 | 1.33 |
|  | 4 | 1.58 |

**Table S4**: CO_2_ adsorption data of 100AlPO_4_-5 and 400AlPO_4_-5 at various pressures and at 25 °C.

| **Sample** | **Pressure (bar)** | **CO_2_ adsorption capacity (mmol CO_2_/g)** |
| --- | --- | --- |
| 100AlPO_4_.5 | 0 | 0 |
|  | 1 | 0.52 |
|  | 2 | 1.01 |
|  | 3 | 1.36 |
|  | 4 | 1.57 |
| 400AlPO_4_.5 | 0 | 0 |
|  | 1 | 0.26 |
|  | 2 | 0.54 |
|  | 3 | 0.75 |
|  | 4 | 0.89 |

**Table S5**: Maximum CO_2_ adsorption capacity of 100AlPO_4_-5 up to 4 bar (experimental and model-calculated) at different temperatures.

| **Temperature (**°C) | **Maximum CO_2_ adsorption capacity at 4 bar (mg CO_2_/g) -Experimental** | **Maximum CO_2_ adsorption capacity at 4 bar (mg CO_2_/g) - Model-calculated** |
| --- | --- | --- |
| 25 | 66.9 | 65.7 |
| 45 | 50.1 | 46.8 |
| 60 | 32.5 | 30.9 |

**Supporting Figures:**

**Figure S1.** Physical appearance of AlPO_4_-5 crystals calcined at (a) 300 °C, (b) 400 °C, (c) 500 °C, (d) 600 °C and (e) 700 °C

**
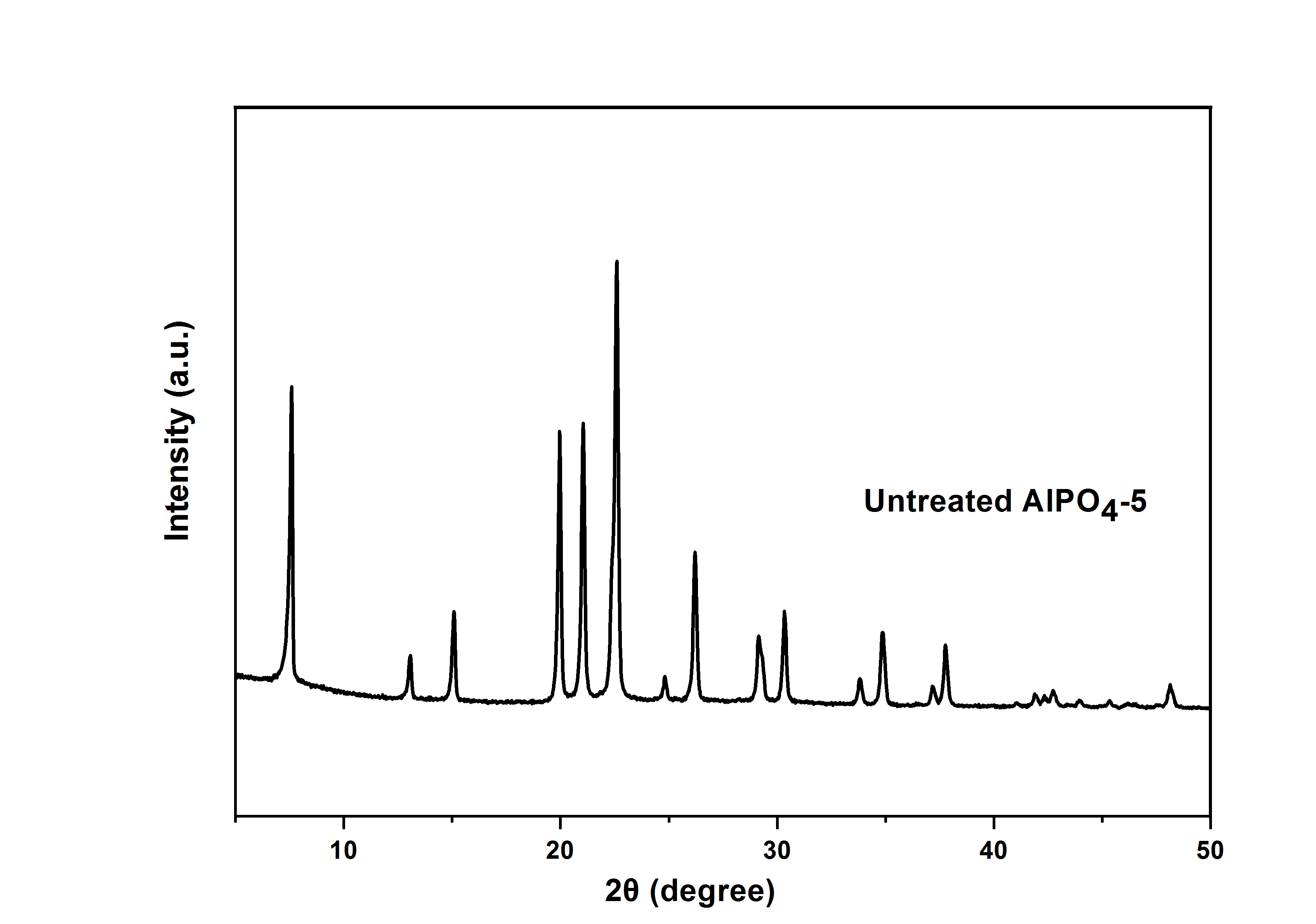
**

**Figure S2.** XRD pattern of as-synthesized, untreated AlPO_4_-5


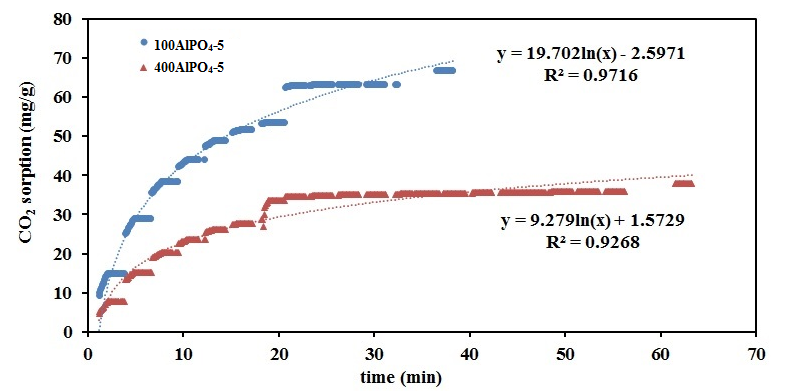


**Figure S3:** Time on stream CO_2_ adsorption data for 100AlPO_4_-5 and 400AlPO_4_-5 at 25 ^o^C and up to 4 bar.


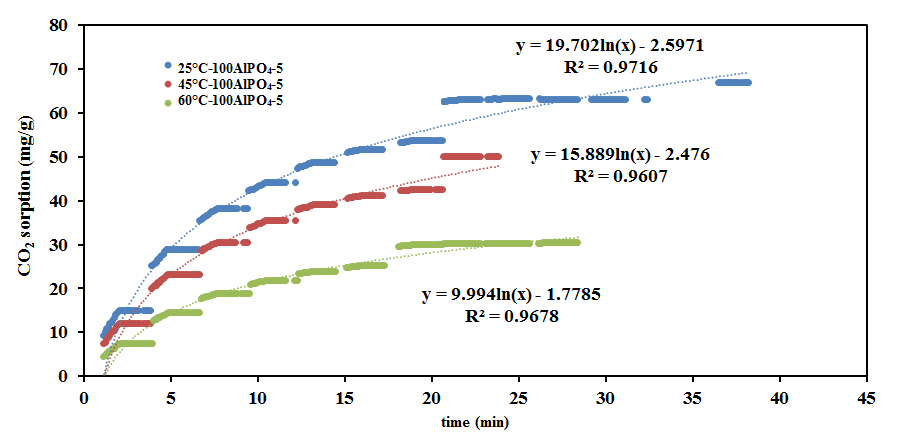


**Figure S4:** Time on stream CO_2_ adsorption data of 100AlPO_4_-5 at different temperatures and up to 4 bar.

**(a)**

**(b)**

**(c)**

**Figure S5:** Linear Lagergren model fitting for calculation of adsorption rate constant k up to 4 bar at (a) 25 ^o^C, (b) 45 ^o^C, and (c) 60 ^o^C.
